# MicroRNA-224 Promotes Tumorigenesis through Downregulation of Caspase-9 in Triple-Negative Breast Cancer

**DOI:** 10.1155/2019/7378967

**Published:** 2019-02-11

**Authors:** Li Zhang, Xin Zhang, Xin Wang, Miao He, Shixing Qiao

**Affiliations:** ^1^Department of Anesthesia, The Second Hospital of Jilin University, Changchun, Jilin, China; ^2^Department of Hepatopancreatobiliary Surgery, The Second Hospital of Jilin University, Changchun, Jilin, China

## Abstract

Triple-negative breast cancer (TNBC) harbors genetic heterogeneity and generally has more aggressive clinical outcomes. As such, there is urgency in identifying new prognostic targets and developing novel therapeutic strategies. In this study, miR-224 was overexpressed in breast cancer cell lines and TNBC primary cancer samples. Knockdown of miR-224 in MDA-MB-231 cancer cells reduced cell proliferation, migration, and invasion. Through integrating in silico prediction algorithms with KEGG pathway and Gene Ontology analyses, *CASP9* was identified to be a potential target of miR-224. miR-224 knockdown significantly increased *CASP9* transcript and protein levels. Furthermore, luciferase reporter assays confirmed a direct interaction of miR-224 with *CASP9*. Our findings have demonstrated that the miR-224/CASP9 axis plays an important role in TNBC progression, providing evidence in support of a promising therapeutic strategy for this disease.

## 1. Introduction

Triple-negative breast cancer (TNBC) is a subset of breast cancer and is characterized by the negative expression of human epidermal growth factor receptor 2 (HER2), estrogen (ER), and progesterone (PR) receptors [1]. TNBC accounts for 10-20% of invasive breast cancer and is more common in young women, exhibiting more aggressive clinical behavior and distinctive metastatic patterns [2–4]. Due to the absence of specific molecular markers, the current major treatment for early-stage or recurrent TNBC remains chemotherapy with or without radiotherapy. Although PARP and EGFR-TKI inhibitors have been tested in the patients with TNBC, their clinical benefits are still uncertain, resulting in poor prognosis [4]. Therefore, there is an urgent need to identify the prognostic biomarkers of TNBC to distinguish patients and allow selection of suitable patients for therapeutic opportunities.

MicroRNAs (miRNAs) are small noncoding RNA molecules that posttranscriptionally regulate the expression of multiple genes [5]. Increasing evidence suggests that miRNAs can function either as oncogenes or tumor suppressors [6] and play a pivotal role in cancer prognostic, predictive, diagnostic, and therapeutic avenues [7, 8]. In TNBC, a number of miRNAs, such as miR-221, miR-21, miR-210, miR-10b, miR-145, miR-205, and miR-122a, have been identified and their expressions are significantly different between cancer and normal tissues [9]. Furthermore, miRNA downstream target genes may be involved in many critical cellular functions, leading to regulations in tumor invasion and migration [10, 11]. Recently, a serum signature of four miRNAs (miR-18b, miR-103, miR-107, and miR-652) in TNBC was found to be associated with a good clinical outcome [12]. Therefore, developing microRNA-based therapeutics might improve cancer treatment, particularly for TNBC patients who demonstrate early relapse and poor overall survival.

Certain observations regarding the function of miR-224 in breast cancer have been controversial. For instance, in the mouse xenograft model from the breast cancer cell line MCF-7, the ectopic expression of *Ubc9* caused the downregulation of miR-224, which in turn, increased cell invasion [13]. In contrast, miR-224 was significantly upregulated in the highly invasive MDA-MB-231 cells and was associated with the repression of the stroma-associated RKIP target genes, *CXCR4, MMP1*, and *OPN*, all of which are involved in breast tumor metastasis to the bone [14]. These data emphasize the need for investigations into the causative mechanisms of miR-224 in TNBC.

In this present study, we have reported the overexpression of miR-224 in breast cancer, particularly in the TNBC cell lines and primary cancer tissues. We assessed the cellular and molecular functional changes upon the knockdown of miR-224. Furthermore, we have identified one of its direct and key targets—*CASP9*—which in turn, mediates tumor proliferation, migration, and invasion through cell apoptosis pathways, providing evidence in support of targeting miR-224/*CASP9* in TNBC therapy.

## 2. Materials and Methods

### 2.1. Cell Lines and Patient Samples

Triple-negative breast cancer cell lines MDA-MB-231 and MDA-MB-468, as well as the Luminal A breast cancer cell lines T47D and MCF-7, were cultured in RPMI 1640 supplemented with 10% fetal bovine serum. Human normal mammary gland epithelial cells MCF-10A cells were grown in DMEM/HAM's F12 supplemented with 5% horse serum, insulin, and hydrocortisone. All cells were maintained in a 37°C incubator with a 5% CO2 humidity. Twenty-two TNBCs and eleven Luminal A/B subtypes of breast cancer samples were collected; and twelve available adjacent normal tissues were served as normal comparators. This study received the Research Ethics Board approval from the Second Hospital of Jilin University.

### 2.2. Cell Transfection Experiments

The biological effects of miR-224 were investigated by transfection of antimiR negative control (NC) and antagomiR-224 using the Lipofectamine 2000 (Invitrogen) reverse transfection protocol. All cells were transfected at a final concentration either of 40 nM or 80 nM based on the experiment design.

### 2.3. Quantification of miRNAs and mRNAs

Total RNA was extracted from either cell lines or primary tissues using the RNA extraction kit from Qiagen or the RecoverAll Total Nucleic Acid Isolation kit for FFPE (Ambion) according to the manufacturer's instructions. The RNA was then reverse transcribed using SuperScript II Reverse Transcriptase (Invitrogen) according to the manufacturer's recommendations. Quantitative real-time PCR (qRT-PCR) analysis was performed using the ABI PRISM 7900HT Sequence Detection System (Applied Biosystems Inc). The expression of hsa-miR-224 (miR-224-5p) was measured using the standard Taqman MicroRNA Assay (Applied Biosystems) as previously reported [15]. RNU48 were used as endogenous controls. The putative miR-224 target gene expressions were measured using qRT-PCR. *GAPDH* was used as an endogenous control. The primers used for mRNA expression are the following: forward: 5′-ctagtttgcccacacccagt-3′ and reverse: 5′-gcattagcgaccctaagcag-3′ for *CASP9*, forward: 5′-accacccaataccacaggaa-3′ and reverse: 5′-cattgggagctgatgaggat-3′ for *BRAF1*, forward: 5′-tcaacaaggagcatgagcac-3′ and reverse: 5′-agtgtgcctttaccccactg-3′ for *TRIM9*, and forward: 5′-gtcggatggtcacctgatct-3′ and reverse: 5′-cccatagccataagcctgaa-3′ for *PLEKHB2*.

### 2.4. Cell Proliferation Assays

The cytopathic effects of MDA-MB-231 cells transfected with antagomiR-224 (antimiR-224) were evaluated using the CellTiter 96 Non-Radioactive Cell Proliferation Assay (MTS) (Promega Biosciences). Cell proliferative activity was measured at 24, 48, and 72 hours after transfection.

### 2.5. Cell Migration and Invasion

The migration and invasive ability of MDA-MB-231 cells were assessed using the BD Biosciences BioCoat Control Chamber and the Matrigel Invasion Chamber. 1 × 10^5^ cells were transfected with either antagomiR-224 or NC and plated on either control inserts (PET membrane) or transwell chambers precoated with Matrigel. The medium containing 15% fetal bovine serum in the lower chamber served as the chemoattractant. After 24 hours' incubation, noninvading cells were removed from the upper surface of the membrane, with cotton swabs. The invasive cells attached to the lower surface of the membrane insert were then fixed and stained with a Diff-Quick Stain (BD Biosciences). The number of invasive cells was counted under a microscope.

### 2.6. Caspase-3/7 Activities

The antagomiR-224- or NC-transfected MDA-MB-231 cells were seeded onto 96-well plates (5 × 10^3^ cells per well). After 48 or 72 hours of incubation at 37°C, caspase-3/7 substrates were added according to the manufacturer's specifications. Caspase-3/7 activities were then analyzed using the SPECTRAFluor Plus Fluorometer.

### 2.7. Western Blotting

MDA-MB-231 cells (5 × 10^5^ cells per well) were transfected with either antagomiR-224 or NC 72 hours posttransfection; the cells were collected and lysed. Protein extracts were prepared and quantified using the BCA method. 20 *μ*g of protein were loaded onto 10%Tris-glycine protein gels and then transferred onto a nitrocellulose membrane. The membranes were blocked in 5% milk in Tris-buffered saline with 0.1% Tween-20 (TBST) and probed with rabbit polyclonal anti-*Caspase-9* (Sigma-Aldrich, 1/500) and *GAPDH* (Abcam, USA) antibodies overnight and was followed by incubation with the second antibodies (Abcam, USA) labeled with horseradish peroxidase for 2 hours. Signals were visualized using the ECL Western Blotting Substrate kit (Pierce, USA).

### 2.8. Luciferase Assay

To assess the direct targets of miR-224, two selected genes *CASP9* and *BRAF1*, which have putative miR-224 binding sites in their 3′-UTR regions, were amplified by PCR and cloned downstream of the firefly luciferase gene in a pMIR-REPORT luciferase vector (Ambion). A mutant sequence was also cloned as a validation plasmid. Either empty or pMir-luciferase-gene specific vectors were cotransfected with antagomiR-224 or NC in MDA-MB-231 cell lines. As reference control, pRL-SV vector (Promega) containing Renilla luciferase was also transfected to each well. Dual-Luciferase Reporter Assay (Promega) was used to assess both the firefly and Renilla luciferase activities.

### 2.9. Statistical Analysis

Box plots were utilized to visually explore the expression of miR-224 or their target genes using a Mann-Whitney-Wilcoxon test. All other data were expressed as the mean ± SE; a *p* value of <0.05 was considered to be statistically significant.

## 3. Results

### 3.1. Overexpression of miR-224 in Triple-Negative Breast Cancer

**Figure 1:**
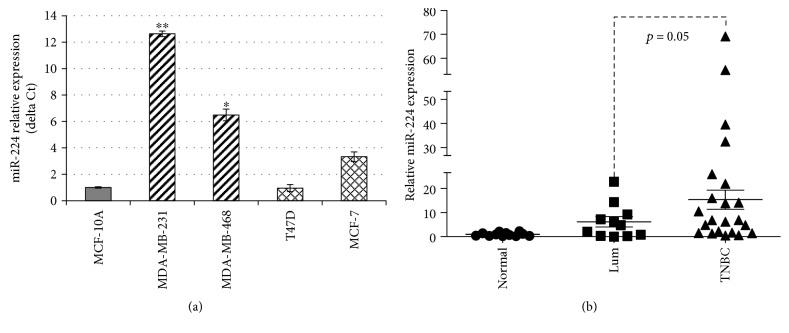
Overexpression of miR-224 in triple-negative breast cancer. (a) qRT-PCR analysis of miR-224 expression in breast cancer cell lines: MDA-MB-231, MDA-MB-468, T47D, and MCF-7, compared with the MCF-10A cell line; ^∗^ *p* < 0.05 and ^∗∗^ *p* < 0.01. (b) Levels of miR-224 expression were measured in a group of breast cancer patient samples, including 11 luminal and 22 TNBC subtypes using quantitative real-time PCR.

To assess differential expression of miR-224 in breast cancer, two triple-negative breast cancer cell lines MDA-MB-231 and MDA-MB-468 and two luminal cell lines T47D and MCF-7 were used in this study. miR-224 was overexpressed in most breast cancer cell lines when compared to a normal mammary gland epithelial cell MCF-10A cells. Of note, miR-224 exhibited a higher expression level in TNBC than in luminal cell lines (Figure 1(a), ^∗^ *p* < 0.05 and ^∗∗^ *p* < 0.01).

The expression level of miR-224 was further examined in a group of breast cancer patient samples including 11 luminal and 22 TNBC subtypes. miR-224 expression was elevated in most patient cancer specimens; however, the expression level was significantly higher in TNBC patients than that in luminal-type patients (Figure 1(b), *p* = 0.05). For the sample which has available adjacent normal tissue, the relative expression of miR-224 in cancer versus the adjacent normal tissue for each individual case is summarized in the Supplementary Table (available here).

### 3.2. miR-224 Downregulation Significantly Reduced Cell Proliferation, Migration, and Invasion

**Figure 2:**
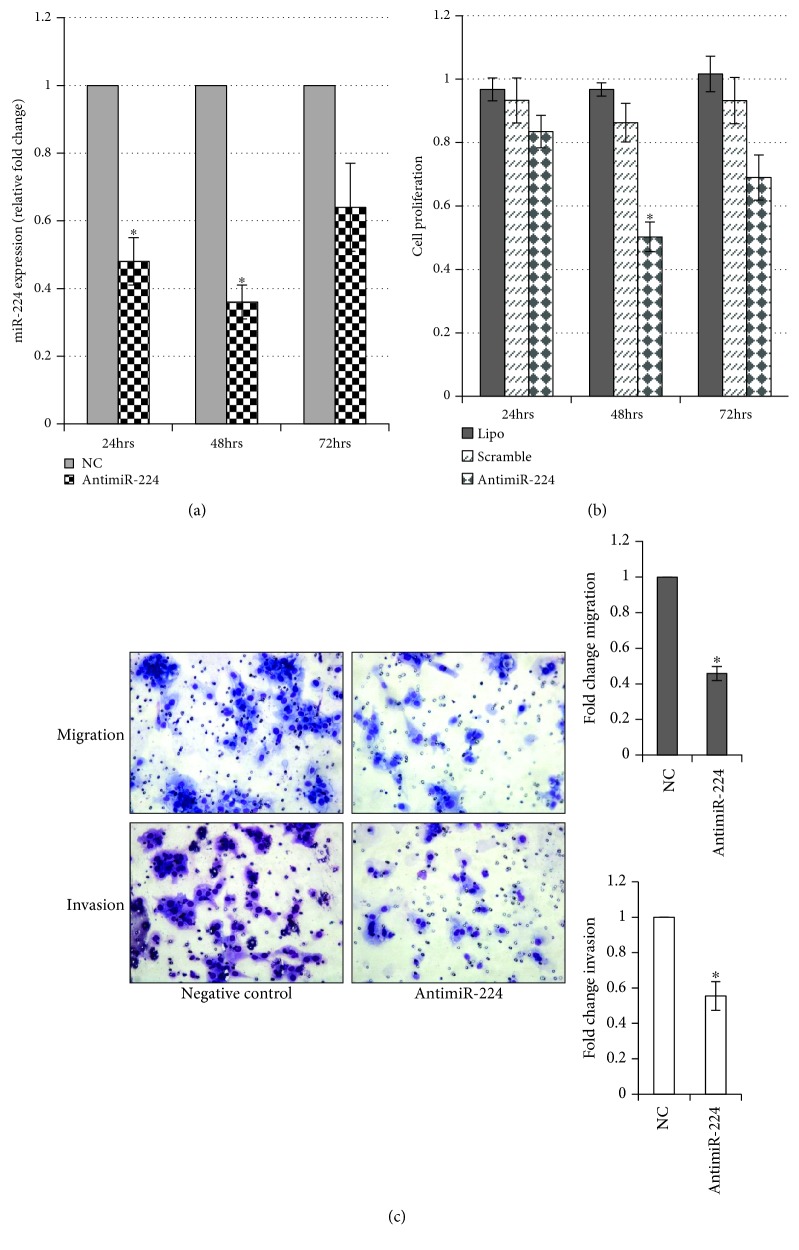
*In vitro* effects of the downregulation of miR-224. (a) AntagomiR-224 was transfected into MDA-MB-231 cells, and miR-224 expression was measured at 24, 48, and 72 hours posttransfection; ^∗^ *p* < 0.05. (b) AntimiR-224 significantly reduced MDA-MB-231 cell proliferation at 48 and 72 hours posttransfection, compared to the scramble control antimiR (40 *μ*M), using the MTS assay; ^∗^ *p* < 0.05. (c) Representative images and quantification depicting the reduction of migratory ability (top) and invasion (bottom) of MDA-MB-231 cells that were transfected with 40 *μ*M of antimiR-224 compared to the negative control antimiR. All data represent the mean ± SE from 3 independent experiments; ^∗^ *p* < 0.05.

The biological significance of miR-224 was assessed in cancer cells. MDA-MB-231 cells were transfected with 40 *μ*M of negative control or antimiR-224. The reduction in miR-224 expression level started as early as 24 hours after transfection and further reduced by 38% at 48 h, which persisted until 72 hours posttransfection (Figure 2(a)). This reduction in miR-224 expression led to a significant decrease in the cell viability by 50% at 48 h and 68% at 72 hours compared to that in the control (Figure 2(b)). Additionally, suppression of miR-224 resulted in a significant reduction in the cell migration (42%) and invasion (55%) of MDA-MB-231 cells compared to that of the negative control (Figure 2(c)).

### 3.3. Identification of Direct Targets of miR-224

**Figure 3:**
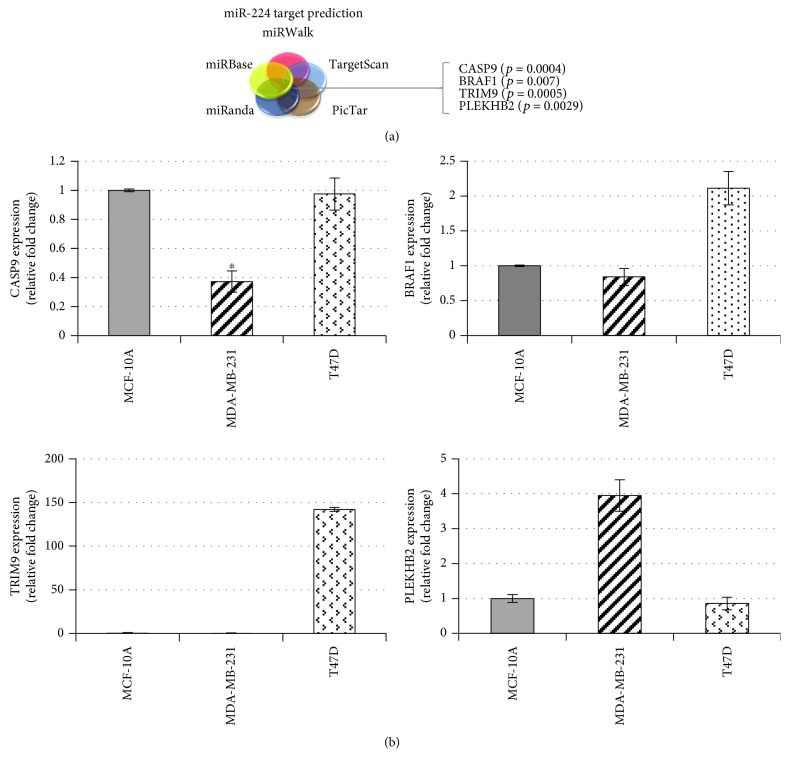
Putative miR-224 targets identification. (a) Five in silico microarray datasets were utilized, and Kyoto Encyclopedia of Genes and Genomes (KEGG) pathway and Gene Ontology (GO) enrichment analyses were performed. (b) Baseline expressions of putative miR-224 targets (*CASP9, BRAF1, TRIM9,* and *PLEKHB2*) were analyzed by qRT-PCR. ^∗^ *p* < 0.05.

In our *in vivo* and *in vitro* studies, miR-224 appeared to be an important mediator in breast cancer, particularly in TNBC progression. In order to investigate putative mRNA targets of miR-224, five in silico publicly available microarray datasets were utilized. This method identified 11 potential downstream targets of miR-224. To further study the involvement of these genes in essential cellular processes and/or tumor progression, Kyoto Encyclopedia of Genes and Genomes (KEGG) pathway and Gene Ontology (GO) term enrichment analyses were performed. Using these approaches, four overlapping transcripts were selected for validation: *CASP9, BRAF1*, *TRIM9,* and *PLEKHB2* (Figure 3(a)). The baseline expression of each gene was examined by individual qRT-PCR assays. The results demonstrated that *CASP9* and *BRAF1* were indeed underexpressed in MDA-MB-231 cells comparing to those in MCF-10A normal mammary gland epithelial cells, but they were either equally expressed or overexpressed in T47D cells (Figure 3(b)). These findings indicated that both *CASP9* and *BRAF1* are highly probable targets of miR-224 in TNBC cells.

### 3.4. miR-224/CASP9 Axis-Regulated Cell Apoptosis through Caspase-3/7 Activation

**Figure 4:**
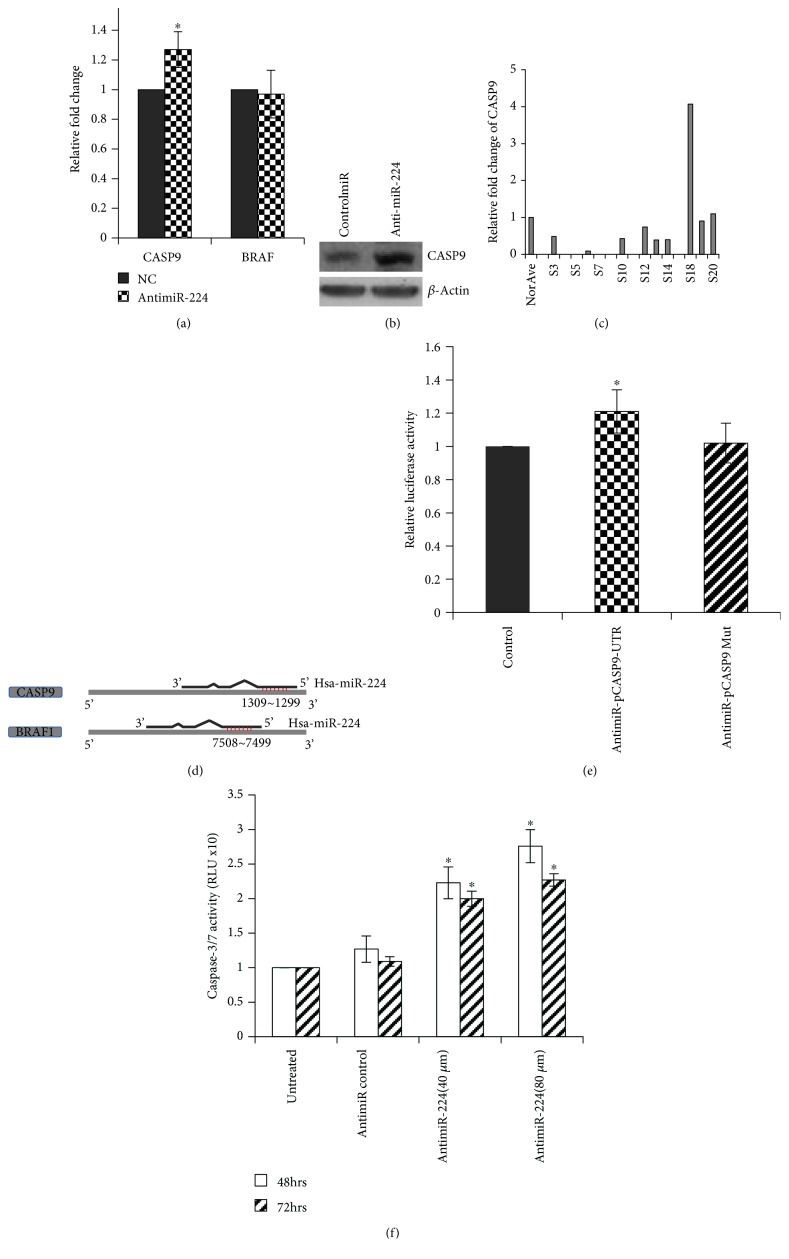
*CASP9* is directly targeted by miR-224. (a) *CASP9* transcript expression in MDA-MB-231 cells was measured 48 hours posttransfection with antimiR-224 (40 *μ*M) or negative control miR (40 *μ*M). (b) Western blotting of *CASP9* in MDA-MB-231 cells was performed 72 hours posttransfection. (c) *CASP9* expression was measured in TNBC samples. (d) Schema shows miR-224 interaction with its target mRNAs—*CASP9* and *BRAF1*. miRNA seed sequence was shown in red. (e) Luciferase reporter assay of MDA-MB-231 cells cotransfected with pCASP9-UTR or pCASP9 Mutant plasmids and either antimiR-224 or negative control miR. Samples were analyzed 72 hours posttransfection, and data were normalized to the pmiR plasmid only transfection. Data represents the mean ± SE; *n* = 3 and ^∗^ *p* < 0.05. (f) Caspase-3/7 activities were measured at 48 and 72 hours posttransfection with untreated, antimiR control, antimiR-224 (40 *μ*M/well), or antimiR-224 (80 *μ*M/well). Each datum represents the mean ± SE from 3 independent experiments.

To identify the direct targets of miR-224, qRT-PCR assays were used to validate *CASP9* and *BRAF1* transcript levels in MDA-MB-231 cells posttransfection of antimiR-224. As shown in Figure 4(a), the level of *CASP9* increased significantly after miR-224 knockdown compared to its level in the negative control (NC), while the level of *BRAF1* did not change. Western blotting showed an increase in the expression of *CASP9* after knockdown of miR-224 (Figure 4(b)). In the available TNBC tissue samples (*n* = 15), the relative expression of *CASP9* was lower in 7 out of 9 samples in comparison to that in the adjacent normal tissues, while the other six samples showed indeterminable value of *CASP9 (* Figure 4(c)).

In order to establish a direct interaction between miR-224 and the 3′-UTR of *CASP9*, a luciferase assay was employed. A luciferase constructed plasmid was designed and made for *CASP9*.  Figure 4(d) illustrates the overlapping seed sites of miR-224 with *CASP9* and *BRAF1*. MDA-MB-231 cells were cotransfected with antimiR-224, and pMIR-REPORT-CASP9 and luciferase activity was measured and compared with those in cells cotransfected with NC. The cells cotransfected with both antimiR and pMIR-REPORT-CASP9 demonstrated a significant increase in luciferase activity compared to the control cells. This effect was abrogated after mutating the 3′-UTR region of *CASP9* (Figure 4(e)).

As sequential activation of caspases plays a central role in cell apoptosis, *CASP9* downstream cascades, the activities of *caspase-3* and *caspase-7* were measured (Figure 4(f)). Cells were transfected with either scramble control or antimiR-224 (40 *μ*M or 80 *μ*M, respectively). After 48 or 72 hours of incubation, the caspase-3/7 activities demonstrated significant increase by 2.2-fold (40 *μ*M) and 2.7-fold (80 *μ*M) in antimiR-224-treated MDA-MB-231 cells, compared to the mock or scramble control cells.

## 4. Discussion

In the current study, we have demonstrated that miR-224 is significantly overexpressed in both TNBC cell lines and primary cancer samples. miR-224 overexpression is also associated with tumor cell proliferation, migration, and invasion. These pleiotropic effects of miR-224 were directly mediated through cellular apoptosis regulation by *CASP9 (caspase-9)*.

The role of miR-224 was first reported by Wang et al. [16]. miR-224 was found to be the most significantly upregulated miRNA in hepatocellular carcinoma patients. miR-224 acted through interaction with apoptosis inhibitor-5 transcript expression to regulate tumorigenesis [16]. Since then, miR-224 has been reported to be deregulated in various human malignancies. It is upregulated in cervical cancer [17], glioma [18], nonsmall cell lung cancer [19], and colorectal cancer [20] and downregulated in prostate cancer [21], indicating a dual regulatory role played by miR-224 either as an oncogene or a tumor suppressor. The molecular function of miR-224 has been studied recently, and a growing body of evidence has shown that miR-224 regulates tumor proliferation through several signal transduction pathways, such as cell cycle progression [22] and NF-*κ*B and TGF-*β* signaling pathways [23, 24], through direct interactions with various tumor-related genes including *PHLPP1* and *PHLPP2* in colorectal cancer [20], *SMAD4* and *TNFAIP1* in NSLC [25], and *TPD52* in prostate cancer [21]. Further studies have suggested that miR-224 can be a treatment indicator for chemoresistance or radiation sensitivity [26, 27]. All of the above findings document the critical role of miR-224 deregulation in cancer progression.

However, the function of miR-224 in breast cancer has been controversial [13, 14]. Our current study found that miR-224 is highly overexpressed in TNBC cancer cells (Figure 1(a)). These phenotypes were further validated in the primary TNBC samples (Figure 1(b)). Moreover, the increase in the expression of miR-224 mediated cell proliferation, migration, and invasion (Figure 2), which further supports an oncogenic role of the ectopic expression of miR-224 in TNBC.

The underlying mechanisms for miR-224 upregulation in TNBC remain unclear. Our study has identified a novel direct target for miR-224, an apoptosis-related cysteine peptidase caspase-9 (*CASP9*). Apoptosis is the most common form of programmed cell death involving the extrinsic (the death receptor) pathway and the intrinsic (mitochondria-mediated) pathway [28, 29]. *CASP9* plays a central role in the mitochondrial pathway, in which it binds to the apoptotic peptidase activating factor 1 (*Apaf-1*) in response to the release of cytochrome c from the mitochondria [30, 31]. The activated *CASP9* then cleaves and activates the effector *caspase-3* and *-7*, leading to apoptosis. However, increasing evidence has shown that the failure to activate *CASP9* under physiological and pathophysiological conditions can result in degenerative and developmental disorders and even cancer [32]. Studies have shown that a variety of endogenous proteins or small molecules are involved in regulating *CASP9* function, such as *ERK2* [33], *Akt/PKB* [34], and *HBXIP* [35]. MicroRNAs engage in regulating RNA molecules; however, only three miRNAs miR-24a, miR-582-5p, and miR-23a have been reported to regulate *CASP9* function in colorectal or glioblastoma cells [36, 37]. In the current study, we have demonstrated direct functional changes of *CASP9* under the regulation of miR-224, which affects cell proliferation, migration, and invasion through the mediation of caspase-3/7 activities.

In conclusion, a novel oncogenic role for miR-224 has been identified in TNBC, in which overexpression of miR-224 suppresses *CASP9* and results in the inactivation of the apoptosis pathway. In turn, this leads to increased breast cancer cell proliferation, migration, and invasion. Our study highlights the importance of the apoptosis pathway for the aggressive behavior of this disease and provides a potential therapeutic strategy for TNBC patients.

## Data Availability

The data that support the findings of this study are available within the article and its supplementary materials.

## Supplementary Materials

Supplementary Materials Supplementary material contains revised articles on microRNA-224 promoting tumorigenesis in triple-negative breast cancer. Supplementary Table: the relative expression of miR-224 in breast cancer versus adjacent normal tissue.
