# Supplementary material for: MicroRNA-224 Promotes Tumorigenesis through Downregulation of Caspase-9 in Triple-Negative Breast Cancer
Source: Dis Markers. 2019 Feb 11;2019:7378967. doi: 10.1155/2019/7378967 (PMC6388334; doi:10.1155/2019/7378967)
Supplement: Supplementary Materials — Supplementary material contains revised articles on microRNA-224 promoting tumorigenesis in triple-negative breast cancer. Supplementary Table: the relative expression of miR-224 in breast cancer versus adjacent normal tissue. [file 7378967.f1.docx]

**Supplementary Table**

The relative expression of miR-224 in breast cancer versus adjacent normal tissue

|  | **Tumor*** | **Adjacent normal*** |
| --- | --- | --- |
| **lum1** | 0.845967 | 1.307241 |
| **lum2** | 4.837828 | 2.662959 |
| **TN1** | 1.544025 | 1.667214 |
| **TN2** | 1.314374 | 2.911219 |
| **TN3** | 16.05399 | 4.872646 |
| **TN4** | 4.888068 | 4.356425 |
| **TN5** | 1.698392 | 1.30205 |
| **TN6** | 13.94934 | 1.337352 |
| **TN7** | 0.508461 | 1.523468 |
| **TN8** | 4.791963 | 1.216134 |
|  |  |  |
| *, Relative fold change;  lum-luminal-type;  TN-TNBC-type. | | |
